# A hidden infection: Racemose neurocysticercosis causing hydrocephalus; a case report

**DOI:** 10.1016/j.ijscr.2022.107477

**Published:** 2022-08-05

**Authors:** Rachel L. Welch, Brooke Bernardin, Ahmed Albayar

**Affiliations:** a Yale College, Yale University, New Haven, CT 06510, USA; b Department of Neurosurgery, Perelman School of Medicine, University of Pennsylvania, Philadelphia, PA 19104, USA

**Keywords:** NCC, neurocysticercosis, CNS, central nervous system, CT, computed tomography, MRI, magnetic resonance imaging, VPS, ventriculoperitoneal shunt, LP, lumbar puncture, WBC, white blood cells, CSF, cerebrospinal fluid, Neurocysticercosis, Racemose, Hydrocephalus, Cyst, Strongyloides, Tapeworm, Case report

## Abstract

**Introduction and importance:**

Neurocysticercosis (NCC) is the most common helminthic central nervous system infection (CNS) in the Western hemisphere and the most common cause of acquired epilepsy worldwide. Due to its relatively prolonged latent period and clinical similarity to other infectious diseases – including bacterial or viral meningitis and other helminthic infections – NCC may be difficult to diagnose, especially for clinicians who rarely encounter it.

**Case presentation:**

This case report discusses a patient with obstructive hydrocephalus and eosinophilic meningitis secondary to racemose NCC. The diagnosis process was initially complicated by the patient's history of pork allergy and absence of radiographic evidence of helminthic CNS infection. Further investigation showed a 4th ventricle multi-cystic lesion causing hydrocephalus which prompted a surgical intervention with a ventriculoperitoneal shunt (VPS) in conjunction with anti-helminthic medical treatment. At 1-year follow-up, the patient has reported recurrence of VPS related complications.

**Clinical discussion:**

Larval cysts typically deposit within the brain parenchyma, making them easily detected on head computed tomography (CT) scans and leading to neurologic sequelae such as epilepsy. In this case, the absence of CT evidence of NCC and the patient's lifelong history of pork allergy slowed the diagnosis process.

**Conclusion:**

Racemose NCC is a rare subset of the disease in which cyst clusters occupy the extra parenchymal space, thereby changing the symptomatic profile and making the cysts more difficult to visualize in imaging studies. In this case, magnetic resonance imaging (MRI) was the best imaging modality to diagnosis extra parenchymal NCC and guide its surgical management.

## Highlights

• NCC causes parasitic infections in the brain and acquired epilepsy.
• Racemose is a rare form of NCC affecting the extra parenchymal space in the brain.
• Making the diagnosis of NCC is challenging, requiring advanced neuroimaging studies.
• This case report describes racemose NCC complicated by obstructive hydrocephalus.
• Ventriculoperitoneal shunt and anti-parasitic medicines were successful treatments.

## 1 Introduction and importance

Neurocysticercosis (NCC) cysts most commonly develop in the brain parenchyma. Clinically, this form of the disease causes acute encephalitis and presents with headaches and seizures. However, cysts can also be deposited in the extra parenchymal space including within ventricles and subarachnoid spaces. Racemose NCC is a rare and severe subset of extra parenchymal disease in which cysts form confluent clusters [1]. This variant is called racemose NCC after the “grape-like” appearance of these clusters. More specifically, these clusters may form within the basal subarachnoid space and appear grape-like on radiography and pathological studies with a lack of scolices [1]. These multi-cystic lesions are indicative of aberrant proliferation of the tapeworm larvae as they sprout new cysts over time that gradually expand as the scolex degenerates [2]. Racemose NCC is associated with decreased response to anti-helminth treatment and increased surgical complication, thus associated with greater morbidity and mortality [1]. This variant can also present with increased intracranial pressure due to widespread meningitis resulting from CSF obstruction and secondary hydrocephalus [2]. 53 % of patients with racemose NCC present with obstruction in the 4th ventricle, followed by 27 % in the 3rd ventricle, 11 % in the lateral ventricle, and 9 % in the aqueduct [3].

The inflammatory reaction to the parasite is particularly robust in children and young women, leading to more severe disease in these populations [4]. Due to the presence of a protective wall, larval cysts may generate a minimal immune response and remain asymptomatic for years [5]. Thus, initial infection may be relatively remote from the clinical presentation. Patients with extra parenchymal disease, approximately 30 % of NCC cases, present with hydrocephalus secondary to outflow obstruction by cysts, eosinophilic meningitis secondary to cyst rupture, or both [6].

## 2 Case presentation

This is a case report in line with the PROCESS and SCARE 2020 criteria [7], [8]. A 22-year-old female with no significant past medical or surgical history presented to the emergency department with a severe, acute-onset headache accompanied by fever, nausea, vomiting, abdominal pain, photophobia, and posterior neck pain with neck stiffness. She denied skin lesions, diarrhea, or seizures. She reported no sick contacts or recent travel but immigrated from central America three years before and reported that children in her home village had “worms.” She lived with her siblings, a dog, and a cat. She reported an episode of jaundice and dark urine one-year prior to her presentation which resolved spontaneously without medical care. She also reported an allergy to pork. Physical examination showed a fever of 100.7 °F and decreased cervical range of motion secondary to pain. Differential diagnosis included: cryptococcal meningitis, syphilis, HSV encephalitis, bacterial meningitis, parasitic infection, and malignancy. Empiric treatment for bacterial meningitis with Ampicillin, Ceftriaxone, Vancomycin, and Dexamethasone was initiated pending the results of lumbar puncture (LP). No abnormalities were detected on non-contrast head CT at the time of admission while LP showed protein elevation to 74 mg/dL and a white blood cell (WBC) count of 243 with 45 % eosinophils, most consistent with eosinophilic meningitis or aseptic meningitis. Peripheral blood counts showed 1.9 % eosinophils. Cerebrospinal fluid (CSF) culture and gram stain at three days showed no growth. CSF cryptococcal antigen, serum cryptococcal antigen, CSF herpes simplex virus antigen, HIV, and RPR were all negative. This presentation was ultimately thought to be most concerning for Strongyloidiasis, Ascariasis, or Hookworm infection with CSF infiltration although peripheral eosinophilia would have been expected. NCC was thought to be less likely given the patient's allergy to pork. The patient improved with supportive care over the course of the hospital stay (4 days) and was discharged home in a stable condition. Antibiotics were discontinued when blood and CSF cultures returned negative. In addition, Strongyloidiasis antibody, stool nuclear amplification test for Strongyloidiasis, stool Ascaris, and stool hookworm eggs were negative.

**Fig. 1:**
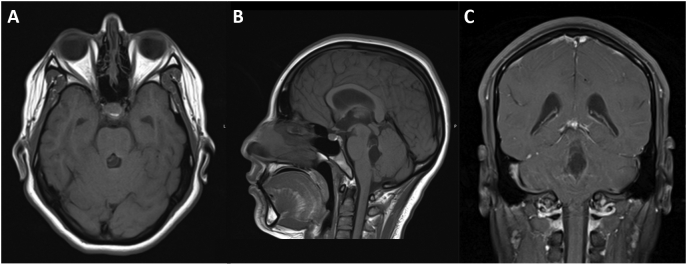
Preoperative Magnetic Resonance Imaging (MRI) scans showing racemose neurocysticercosis (NCC) lesion. (A) Axial pre-contrast MRI T1 scan. (B) Sagittal pre-contrast MRI scan. (C) Coronal post-contrast MRI scan.

**Fig. 2:**
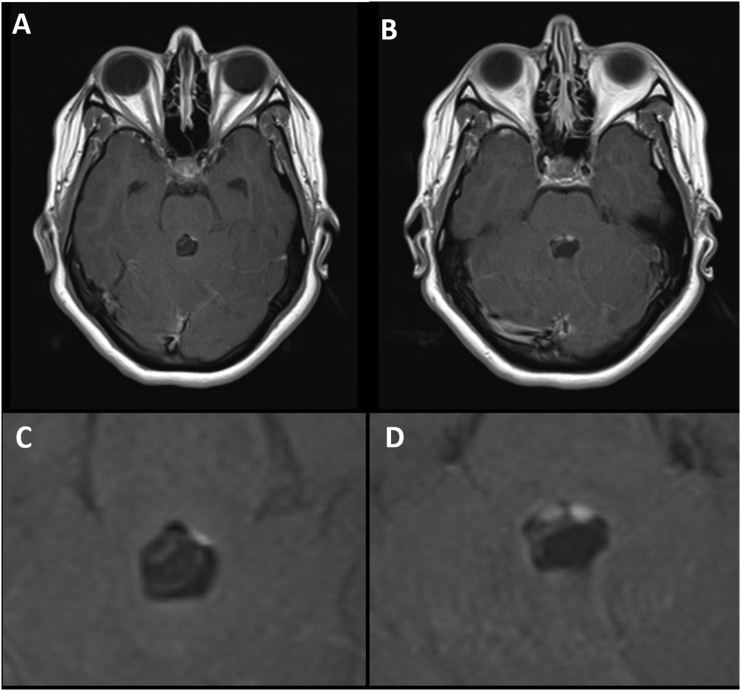
Higher magnification of preoperative Magnetic Resonance Imaging (MRI) axial scans showing racemose neurocysticercosis (NCC) lesion (A, C) pre-contrast, and (B, D) post-contrast.

**Fig. 3:**
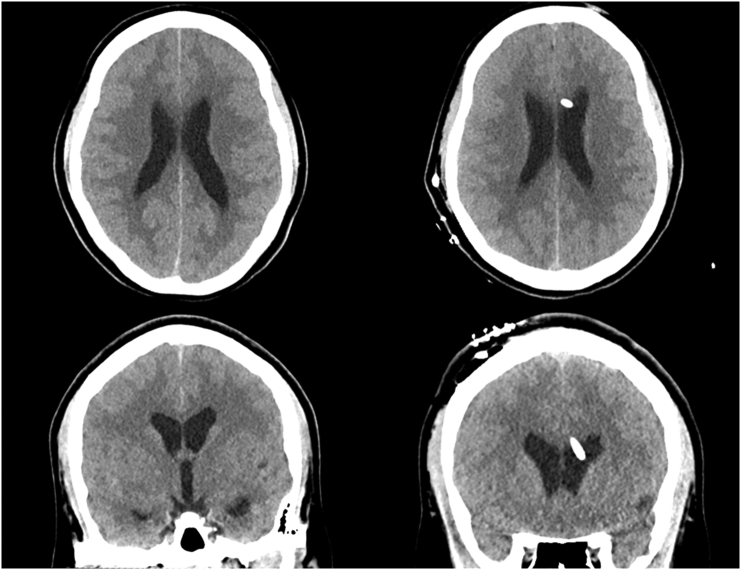
Preoperative vs postoperative head computed tomography (CT) axial scans showing (A) signs of preoperative obstructive hydrocephalus at initial presentation, and (B) the position of a ventriculoperitoneal shunt (VPS) in the eft lateral ventricle.

Two weeks later, the patient presented again to the emergency department with an acute recurrence of her headache, neck stiffness, and associated symptoms from the prior admission. LP was again concerning for eosinophilic meningitis with protein 47 mg/dL and a significantly higher WBC of 420 with 60 % eosinophils. Opening pressure was >35 cm in the seated position. Repeat brain CT showed enlargement of the temporal horns consistent with hydrocephalus and probable hyper densities in the 4th ventricle. Follow-up magnetic resonance imaging (MRI) studies demonstrated a 1.2 × 1.4 × 2.5 cm multi-cystic lesion in the 4th ventricle with a possible scolex along the superior aspect and diffuse prominence of the lateral and 3rd ventricles indicating obstructive hydrocephalus secondary to racemose neurocysticercosis. Fig. 1, Fig. 2 Therefore, a decision was made to place a right side ventriculoperitoneal shunt (VPS) which was performed primarily by the neurosurgery and general surgery team. An adjustable ventricular valve was connected to a right lateral ventricular cannula. The peritoneal tubing was tunneled subcutaneously and was endoscopically placed into the right upper quadrant of the abdomen. Following the surgery, dexamethasone treatment was initiated 24 h before the start of albendazole (1000 mg/day). The patient was discharged on postoperative day 4 in stable condition. Fig. 3 At 6-month and 1-year follow-ups, the patient remained in stable condition without symptom recurrence or VPS complications. Due to the particularly high possibility of VPS occlusion by the cystic debris, MRI evidence of residual infection or hydrocephalus will be used to guide the decision of shunt removal.

## 3 Clinical discussion

NCC can be a difficult diagnosis for multiple reasons. First, misperceptions about the route of transmission and the length of the asymptomatic phase may lead clinicians to miss a possible source in the patient history. For the patient discussed in this case, NCC was most likely acquired from an asymptomatic tapeworm carrier in the household – possibly a family member. However, NCC was placed lower on the differential originally due to the patient's pork allergy and lack of recent travel. The patient's self-limited jaundice and dark urine may represent hemolysis as oncospheres translocated to the brain, suggesting that her NCC infection remained asymptomatic for an entire year.

Additionally, the clinical similarity of extra parenchymal NCC to bacterial meningitis can delay diagnosis and treatment, and blur the clinical picture. Since dexamethasone is a component of treatment for both bacterial meningitis and NCC, empiric treatment of bacterial meningitis may lead to partial symptom relief in patients with NCC and a false belief that the underlying cause of illness had been addressed. This likely occurred in the presented case, leading to the recurrence of symptoms. In retrospect, the clinical symptoms of this patient do match the typical presentation of racemose NCC, with increased intracranial pressure due to meningitis and secondary hydrocephalus [2].

In addition to the treatment of NCC with albendazole and dexamethasone, the presence of obstructive hydrocephalus necessitated the placement of a ventriculoperitoneal shunt. Obstructive hydrocephalus with VPS placement represents a particularly dangerous form of the disease with Colli et al. reporting 30.8 % mortality in patients requiring VPS as well as >80 % shunt failure [9]. Shunt failure is thought to be secondary to proteinaceous debris from cyst rupture obstructing the shunt system. It is important to note, however, that Kelley et al. found a vast benefit for medical anti-helminthic treatment in combination with VPS placement (33 % shunt failure in 6 months) compared to VPS placement alone (90 % failure rate) [10].

Finally, the diagnosis of extra parenchymal NCC was complicated by the use of CT as first-line imaging. CT has lower sensitivity and specificity for detecting intraventricular forms of NCC, as they show radiodensities similar to CSF with only thin membranous walls, hindering proper visualization [2]. However, intraventricular forms can be readily visualized in MRI, especially with 3D heavy T2-weighted sequences with high spatial resolution and signal-to-noise ratio, or a 3D-spoiled gradient echo sequence providing T1 information, which was used to identify the multi-cystic lesions and scolex in this case report Fig. 3 [11].

It is important to note that some authors have reported that racemose NCC typically lacks a scolex [2]. Yet, a scolex was identified in this patient on MRI; therefore, generalizability may be difficult to infer because the patient may be presenting with an atypical or early form of racemose NCC.

We believe that MRI is the best imaging modality in patients with suspected NCC. Further work can be conducted to verify the validity of using MRI as the primary imaging modality for diagnosis, and to track the outcomes of patients treated with a ventriculoperitoneal shunt for symptom relief after surgery.

## 4 Conclusions

In this report, we present a rare case of racemose NCC in a patient with pork allergy which complicated its early diagnosis. In this patient's cases, racemose NCC was complicated with an obstructive hydrocephalus due to the development of a scolex in the 4th ventricle. MRI was the best imaging modality to detect the extra parenchymal lesion in contrast the common reliance on CT imaging. It is important to create a broad differential for meningitis, to include the possibility of NCC and its variants in order to appropriately provide care and treatment as a combination of anti-helminthic therapy and CSF diversion.

## Consent

Written informed consent was obtained from the patient for publication of this case report and accompanying images. A copy of the written consent is available for review by the Editor-in- Chief of this journal on request.

## Sources of funding

This study received no funding or sponsorship.

## Ethical approval

This deidentified case report is exempted by the institutional review board of the University of Pennsylvania, Perelman School of Medicine.

## Provenance and peer review

Not commissioned, externally peer reviewed.

## Author contribution

Rachel L. Welch: Manuscript writing

Brooke Bernardin: manuscript writing and review

Ahmed Albayar: Data collection, Manuscript review

## Registration of research studies

Not applicable.

## Guarantor

Ahmed Albayar.

## Declaration of competing interest

None.
